# The effect of work system on the hand exposure of workers in ^18^F-FDG production centres

**DOI:** 10.1007/s13246-018-0644-9

**Published:** 2018-05-07

**Authors:** Małgorzata Wrzesień

**Affiliations:** Faculty of Physics and Applied Informatics, Department of Nuclear Physics and Radiation Safety, University of Lodz, Pomorska 149/153, 90-236 Lodz, Poland

**Keywords:** Radiopharmaceuticals, Work system, Nuclear medicine, Personnel exposure, Radiation protection

## Abstract

The production of the ^18^F isotope—the marker of deoxyglucose (^18^F-FDG)—the radiopharmaceutical most commonly used in the oncological diagnostic technique of positron emission tomography, requires a cyclotron device. At present, there are nine facilities working in Poland that are equipped with cyclotrons used for producing the short-lived isotopes. The aim of the paper is to determine the hand exposure of workers employed in the two ^18^F-FDG production centres taking in to account the production procedures and work system in those facilities. Measurements, which included all professional workers exposed to ionizing radiation that were employed in two facilities, were performed by using high-sensitivity thermoluminescent detectors during the routine activities of the personnel. The work system used at the production centre has an impact on the level of the recorded doses. Among the production procedures performed by the staff, the highest ionizing radiation doses have been received by the staff during the ^18^F-FDG quality control. The maximum estimated annual Hp(0.07) for chemists from the quality control department can exceed the annual skin limit dose (500 mSv). The source of lowest doses on the hands are the cyclotron operating procedure and the ^18^F-FDG production, provided that these procedures can’t be combined with other production procedures.

## Introduction

^18^F-labeled deoxyglucose (^18^F-FDG), is in wide use in oncological diagnostics via positron emission tomography (PET) technique [1]. The production of ^18^F-FDG is a multistep process that begins by obtaining the ^18^F, and subsequently: labelling the radiopharmaceutical; preparing the individual ^18^F-FDG vial activity; quality control of the resulting compound; packaging the vials with radiopharmaceutical into shielded containers; and transportation of the produced compound to PET departments [2].

Currently in Poland, there are nine centres equipped with a cyclotron for the production of positron-emitting radioisotopes. Polish centres producing radiopharmaceuticals for the purpose of positron emission tomography can be divided taking into account commercial and non-commercial production. Non-commercial production means that the radiopharmaceuticals labeled with short-lived isotopes are solely for the purposes of a PET-CT diagnostic centre (located, in most cases, in the immediate vicinity of the production centre). Commercial production purpose also accounts for the needs of a diagnostic facility located in the immediate vicinity.

In both centre types, the personnel includes physicists, chemists and technical staff. However, in the case of ^18^F-FDG production centres that operate solely for the needs of the nearest PET-CT departments only, nursing staff should also be taken into consideration. The publication presents an analysis of the personnel, as well as the working system in the ^18^F-FDG production centres, in terms of hand exposure to ionizing radiation of the medical staff employed. This work complements the paper by Wrzesień [3] which examined the exposure of the eye lens of workers in radiopharmaceutical production centers.

## Materials and methods

Measurements were carried out in two ^18^F-FDG production centres by using high-sensitivity thermoluminescent detectors (TLDs): LiF: Mg, Cu, P (MCP-N) [4, 5] produced by RADCARD. A gamma radiation source—^137^Cs (^60^Co/^137^Cs irradiator) was used to calibrate the detectors in the Secondary Standards Laboratory in Nofer Institute of Occupational Medicine in Lodz. Dosimeters were calibrated in accordance with ISO 4037-3 [6] in the range from 0.05 to 30 mGy as the air kerma. The H_p_(0.07) for fingers was calculated taking into account the conversion coefficient h_pK_(0.07), given in the ISO International Standard. We used the rod phantoms [7]. The readings of the dosimeters were read out using an RA ‘04 reader from Mikrolab Co.

Measurements were carried out during routine work of the staff in both departments. In the case of the production centre marked RPC I, which produces ^18^F-FDG essentially for commercial purposes, the structure of employment is dominated by a clear division, which in turn is dictated by production procedures performed in the facility. The measurements were carried out for three groups of employees: operators of a cyclotron (two physicists), production workers (three chemists), and quality control staff (two chemists).

Measurements were performed in the span of 3 weeks. The production process at the facility takes place from Monday to Thursday, which represented a total of 11 measurement days. The production, depending on the number of orders, included one or two production runs (shifts) which meant that, during the 11 days of measuring, the ^18^F-FDG was produced 19 times. One shift (production run) represents one vial of the finished product that goes to the quality control laboratory [3]. The activities performed by production workers included the placement of a vial in a synthesizer; the vials were automatically filled with the final radiopharmaceutical product [3].

In the case of the facility producing ^18^F-FDG solely for the needs of a neighboring PET-CT department (marked RPC II), the structure of employment is no longer so heavily dominated by the production procedures carried out in the facility. Here, it is generally easier to distribute the employees based on their specialty training (education). Measurements have covered four chemists, two physicists and four nurses. The task of the physicists was the daily supervision of the proper functioning of the cyclotron. The nurses inject the ^18^F-FDG to the patients in the activity prescribed by the medical doctor. For the chemists, the division of work tasks is not as clearly defined, because the staff were trained so that, in the event of an absence of a worker, another may cover their tasks. This means that, in contrast to the first facility, in the group of chemists, procedures are not strictly assigned to individual people. Thus, all the procedures: production, quality control, and dispensing the dose of radiopharmaceutical for an individual patient at the facility can be done by one person. The production of ^18^F-FDG is carried out in the facility three times a week. The measurements were performed during the 11 days of work.

From the point of view of the occupational structure of the workers employed in RPC II, *Chemist* 1 performs only the combined procedures of the production and dispensing of doses of ^18^F-FDG for patients; alternatively, in addition to the above procedures, quality control of the radiopharmaceutical is carried out as well. *Chemist* 2 performs only the ^18^F-FDG quality control procedures or combines all three procedures—^18^F-FDG production, quality control and dispensing doses of ^18^F-FDG for patients. *Chemist* 3 performs three production procedures or carries out only the procedure for dispensing doses of ^18^F-FDG for individual patients. And the last in the group of *chemists* from the center RPC II—*Chemist* 4 performs only the procedure of dispensing doses of ^18^F-FDG for patients during the second shift. The work specificity of RPC II means that the personnel also includes *nurses* working in shifts.

**Fig. 1:**
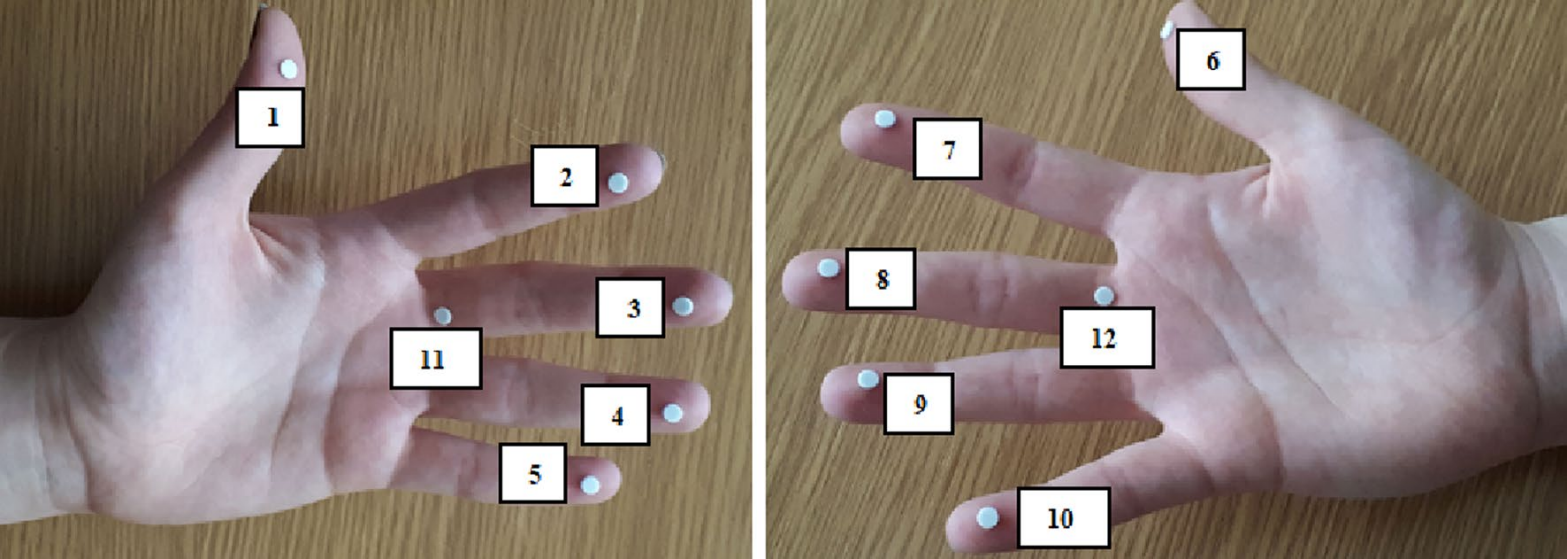
Location of TLDs on worker’s hand. Points ‘11’ and ‘12’ denote points placed at the base of the middle finger of the left and right worker’s hands and corresponding the standard ring dosimeter location

Before the measurements, the detectors were annealed in accordance with the manufacturer’s guidelines and each of them was individually vacuum packed in foil. Thus prepared detectors were placed at the fingertips of the left and right hand and also in a standard ring dosimeter location (both hands) as shown in Fig. 1.

The statistical analysis was performed using the Mann–Whitney test and applying STATISTICA v. 10.0 MR1 software. Any differences found was considered statistically significant if p value was below 0.05.

## Results

### Personnel in radiopharmaceutical production centres (RPC)

**Fig. 2:**
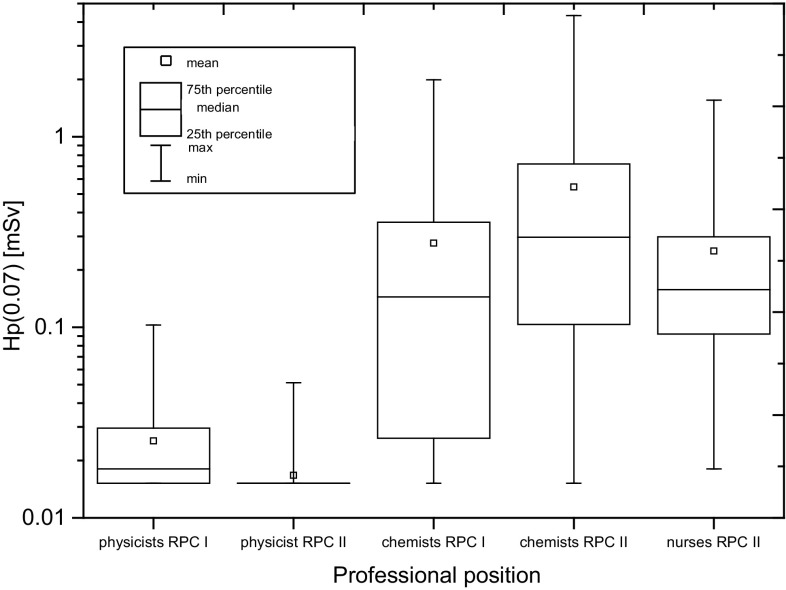
The Hp(0.07) recorded during one working day on hands of the staff employed in radiopharmaceutical production centres, taking into account the professional position of workers. RPCI, RPCII denote, respectively, Radiopharmaceutical Production Centre I and II

Figure 2 presents the values of Hp(0.07) recorded during one working day on the hands of staff employed in radiopharmaceutical production centres, taking into account the professional position of workers.

The greatest differences of Hp(0.07) values recorded during one working day relate to the chemists, in particular those employed in RPC II. This is due to the diversity and spread of production procedures carried out by the same people representing that professional group. This means, therefore, a detailed analysis of individual production procedures carried out in both production facilities taking into account the structure of employment is required.

### Hand exposure during ^18^F-FDG production procedures

**Fig. 3:**
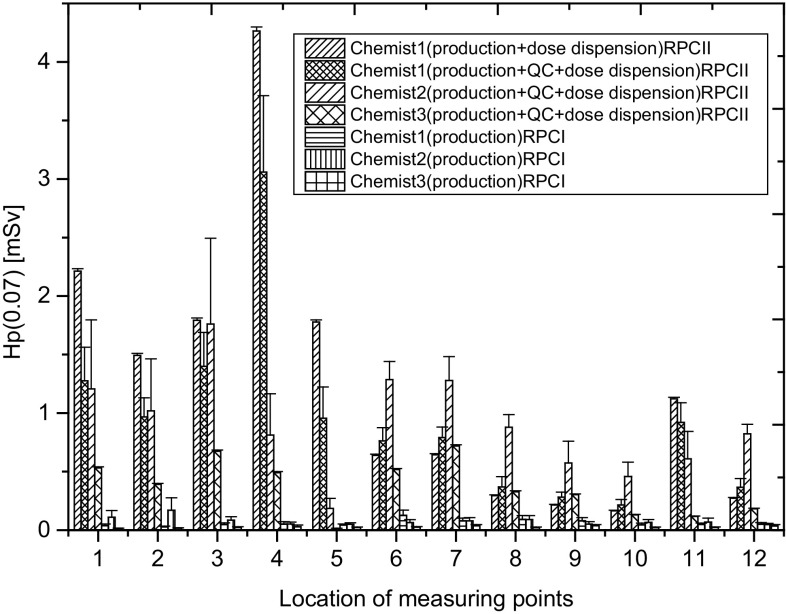
The mean working-day values of Hp(0.07) for staff who performed ^18^F-FDG production procedures in RPC I and RPC II, taking into account the professional position of workers

Figure 3 presents mean working-day values of Hp(0.07) for staff who performed ^18^F-FDG production procedures in RPC I and RPC II, taking into account the professional position of workers.

The highest values of Hp(0.07) recorded during one working day involve basically the chemists employed in RPC II. *Chemists* (1, 2 and 3) employed in RPC I, are performing only the ^18^F-FDG production procedures. In RPC II, employed chemists were performing mostly “cumulative” production procedures, which means that one chemist can perform two or even three radiopharmaceuticals production procedures. During the measurements the *Chemist* 1, *Chemist* 2 and *Chemist* 3 employed in RPC II were performing cumulative procedures, including ^18^F-FDG production and dispensing doses of the radiopharmaceutical, or they jointly performed all three production procedures (^18^F-FDG production, quality control and dispensing doses for patients). The maximum value of Hp(0.07) recorded during one working day for *Chemist* 1 employed in RPC II, who performed cumulative procedures including the production, quality control and dispensing of doses of the radiopharmaceutical was: 4.32 ± 0.07 mSv (for tip of the ring finger of the non-dominant, left hand). In the case of the same worker (*Chemist* 1), who performed two production procedures (^18^F-FDG production, and dispensing dose of radiopharmaceutical for patients) and additionally in the same measuring point, the highest value of Hp(0.07) recorded during one working day was 4.26 ± 0.03 mSv. There are no statistically significant differences between the distributions of Hp(0.07)/A (normalized value of Hp(0.07) to the activity of ^18^F-FDG) recorded for *Chemist* 1 during two production procedures including ^18^F-FDG production and dispensing doses of radiopharmaceutical, and all three production procedures being performed together. At the same time, comparing the distributions of Hp(0.07)/A registered during one working day for three *chemists* carrying out a total of three production procedures (^18^F-FDG production, quality control and dispensing dose of radiopharmaceutical for patients), there was no statistically significant difference. In the case of the RPC I staff—*chemists*, who perform only the ^18^F-FDG production procedure, a statistically significant difference exists between the distribution of Hp(0.07) recorded within one working day for *Chemist* 2 and 3 (p = 0.000037) and *Chemist* 3 compared with *Chemist* 1 (p = 0.000097). Such a difference is not found between the distributions of values Hp(0.07) obtained for *Chemist* 1 and 2 (p = 0.14). In this case the individualized activities performed by individual chemists especially *Chemist* 3 may influence on the level of the Hp(0.07).

### Hand exposure of quality control workers from the shift (production run) point of view

The shift-based working system in the first production facility (RPC I) makes it possible to check the influence of the order of the shifts of quality control employees on the level of the recorded dose.

**Fig. 4:**
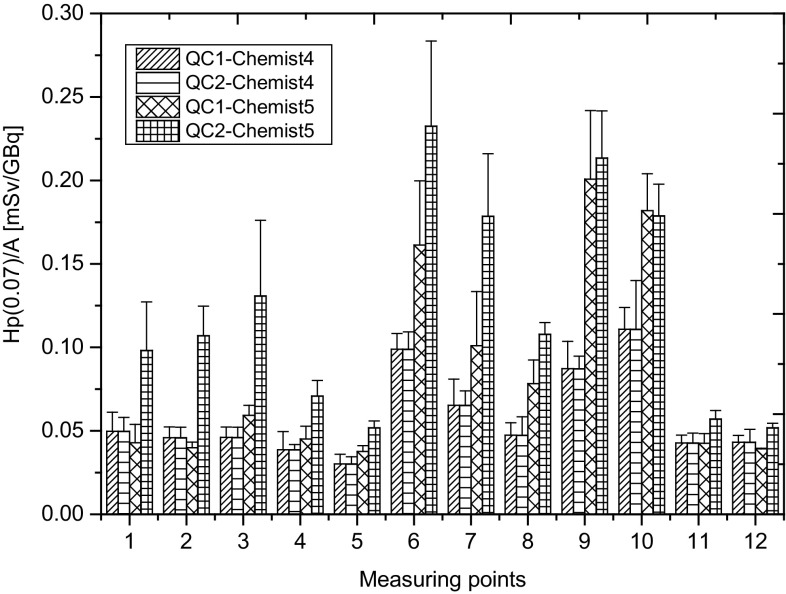
The average values of H_p_(0.07)/A for quality control staff taking into account the production run. QC1 and QC2 denoted the order of the shift (production run) in radiopharmaceuticals quality control department

Figure 4 shows that the trend of the higher fingertips exposure during the second shift is preserved only for the *Chemist* 5. In the case of the *Chemist* 4, the fingertips of the left hand (except the little finger) receive higher doses during the first working shift. The most important factor in this case seems to be the activity of the radiopharmaceutical forwarded to the quality control department. During the first shift, the average ^18^F-FDG activity was 6.4 GBq, for employees of the second shift it was 6.7 GBq [2].

**Fig. 5:**
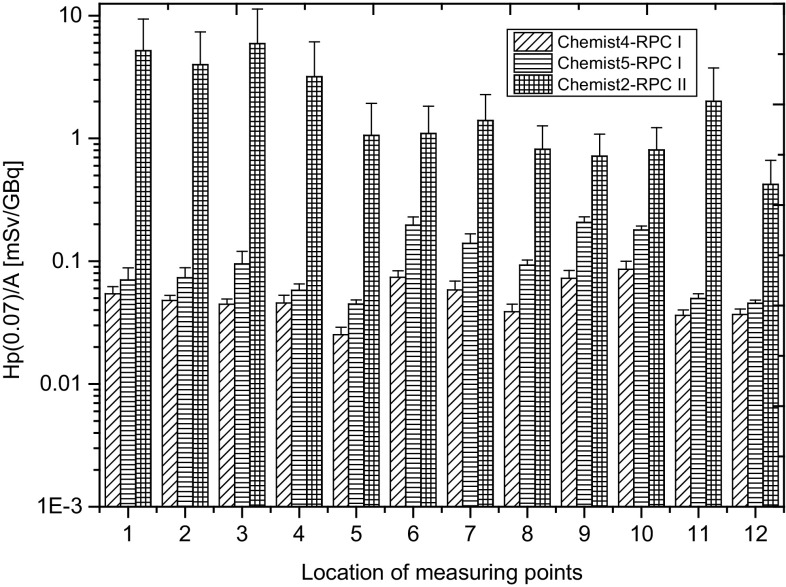
Distribution of mean values of Hp(0.07)/A on the left and right hand two employees of the quality control department at RPC I and one employee in RPC II

In the production facility RPC II, there is no clear assignment of the employee to a specific production procedure; however, it was possible to isolate the quality control procedure in the case of one employee. The comparison of average values Hp(0.07) normalized by the activity in the case of employees of the quality control lab in two production centres is shown in Fig. 5.

It is worth noting that the highest Hp(0.07)/A was recorded for the worker from RPC II—centre, which produces the ^18^F-FDG solely for the use of a neighboring PET-CT department.

Even though the quality control procedures of radiopharmaceuticals produced in both production centres are similar, the mean values of Hp(0.07) normalized by activity, particularly in the case of the left hand of RPC II worker, are at least one order of magnitude higher compared to the two workers from RPC I.

### Hand exposure of workers performing the cyclotron operators procedures against the professional position

**Fig. 6:**
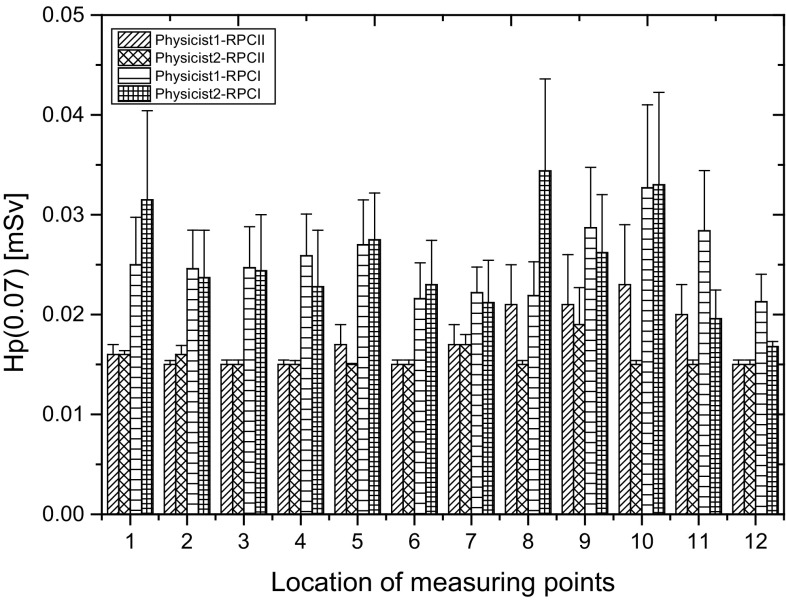
Mean values of Hp(0.07) recorded by the individual measuring points of cyclotron operating personnel in both radiopharmaceuticals production centres

Operating of the cyclotron is a procedure during which the values of Hp(0.07) for employees from the RPC I are, on average, higher than those recorded by the detectors placed on the hands of the staff from RPC II who performed the same procedure (Fig. 6). In terms of the task associated with the operation of the cyclotron, the activities carried out by *physicists* working in RPC II and *physicists* from the RPC I are different.

### Hand exposure of workers during dispensing doses of ^18^F-FDG for patients and injection radiopharmaceuticals to the patients

**Fig. 7:**
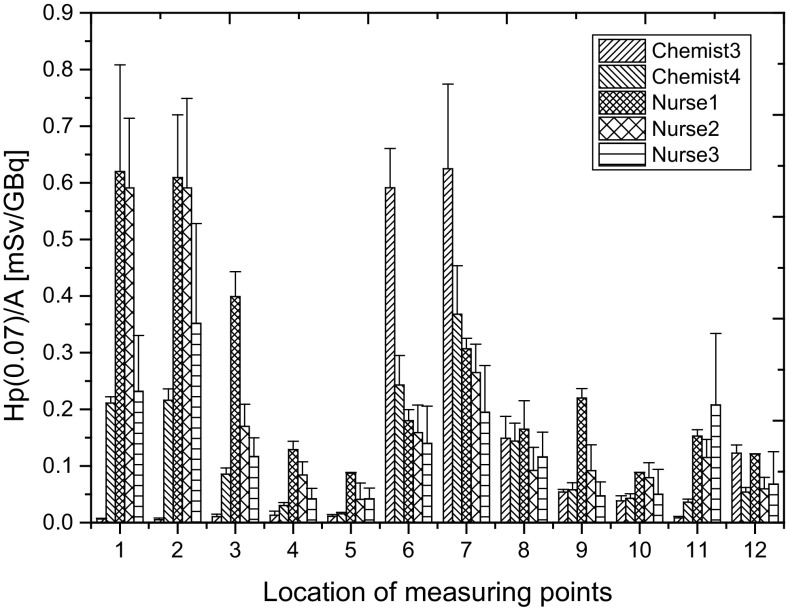
Mean values Hp(0.07)/A recorded by the individual measuring points of chemists who dispense doses of ^18^F-FDG for patients and nurses performing injection of the radiopharmaceutical. All workers are employees in RPC II

Individual dispensing of doses of ^18^F-FDG for patients was performed by two chemists. Figure 7 presents the mean values Hp(0.07)/A for the two *chemists* performing only the dispensing doses of ^18^F-FDG for patients and three *nurses* performing the injection of ^18^F-FDG to the patients. All workers are employees in the RPC II.

The statistically significant difference between the distributions of normalized Hp(0.07) obtained during one working day for *Chemist* 3 and 4 for measuring point 1, 2 and 3 and also 11 and 12 was found (p = 0.018904). Comparing the distributions of mean values of Hp(0.07)/A for *chemists* and *nurses*, a statistically significant difference was found for *Chemist* 3 and *Nurse* 1 (p = 0.014138) and *Chemist* 4 and *Nurse* 1 (p = 0.035090). *Nurse* 1 employed at RPC II works ad hoc solely in case of the absence of *Nurse* 2 or 3. It is true that differences in the distributions of Hp(0.07)/A recorded for three *nurses* can be seen primarily in the case of the tip of the thumb, as well as index and middle fingers of the left hand.

**Fig. 8:**
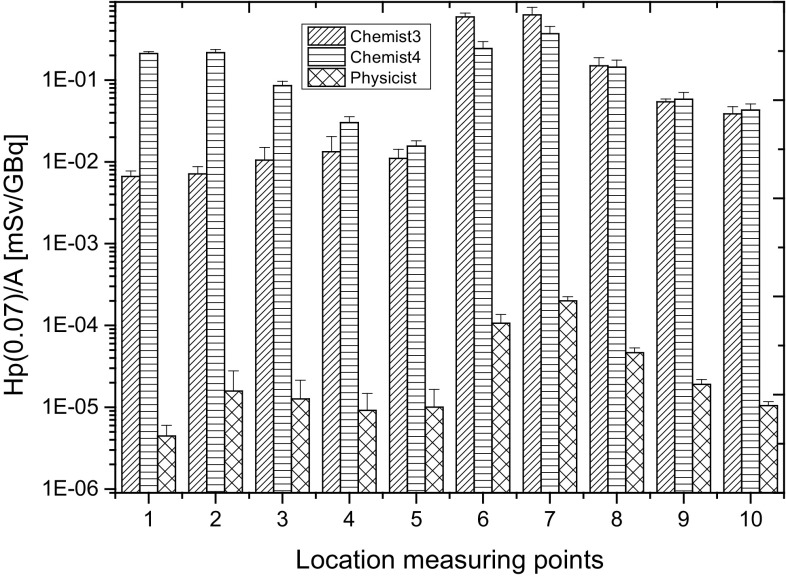
Distribution of Hp(0.07)/A recorded by TLD placed at the fingertips of the left hand and right hand during the dispensing doses of ^18^F-FDG for patients carried out by the chemists employed in the RPC II center and physicist employed in Medical Diagnostic Centre (MDC)

The data for *Chemist* 3 and 4 during the dispensing of doses of ^18^F-FDG for patients was compared with the data from another diagnostic department, where this procedure is realized by a physicist. The total daily ^18^F-FDG activity prepared for patients by a *physicist* is in the range 0.68–2.52 GBq and in the case of *chemists*: 0.83–3.12 GBq. Figure 8 shows the distribution of Hp(0.07)/A recorded by TLD at the fingertips of the left hands and right hands during the dispensing of doses of ^18^F-FDG for patients carried out by *chemists* working in the center RPC II and *physicist* employed in the Medical Diagnostic Centre (MDC) in Lodz provided with PET/CT unit [8].

The average number of patients diagnosed during 1 day in two centres is similar (ten patients), the value of the ^18^F-FDG activity dispensed for a single patient is also approximately the same.

### Estimated annual dose for the workers in ^18^F-FDG production centres

In the case of employees of RPC I, the maximum annual Hp(0.07) was estimated assuming the implementation of 300 production procedures, taking into account only the most exposure fingertips. Annual fingers exposure to ionizing radiation in case of RPC II employees, was estimated taking into account the nature of professional’s work. It was assumed that ^18^F-FDG production took place three times a week (120 working days a year in total). Estimated values are presented in Table 1.

**Table 1:** Maximum estimated annual Hp(0.07) for the staff employed in RPC I and RPC II

Professional groups/RPC	Maximum estimated annual Hp(0.07) (mSv)
Physicists/RPC I	11
Chemists/RPC I	445
Physicists/RPC II	3
Chemists/RPC II	**512**
Nurses/RPC II	135

A bold font was used to emphasize that the maximum estimated annual Hp(0.07) for chemists from the quality control department exceed the annual skin limit dose—500 mSv

## Discussion

The production of radiopharmaceuticals based on short-lived isotopes arouses much interest from the point of view of exposure of personnel performing various production procedures despite the automation of some production procedures. Exposure of personnel in nuclear medicine facilities during the labeling of radiopharmaceuticals by using even ^99m^Tc shows that the main source of exposure of personnel is the same process of radiopharmaceuticals labeling [9]. The ORAMED project [10, 11] discusses in detail the exposure of medical personnel performing procedures using the isotope ^18^F as well. In this case, however, it analyzed the exposure of workers performing manual procedures of labeling the radiopharmaceuticals. The exposure of workers from two ^18^F-FDG production centers discussed in the paper, taking into account both the professional position, as well as the specificity of the work in the facility, suggests that in this area there are also issues that require careful analysis, taking into account the work system of production centres.

The RPC I implements the philosophy of ^18^F-FDG production based on the assignment of specific procedures for employees while taking into account the shift working system. This means that the person performing the various procedures involving cyclotron operation, ^18^F-FDG production and quality control of the finished radiopharmaceutical, varies depending on the order of duty resulting from a schedule roster. In the RPC II, producing ^18^F-FDG solely for the purpose of a PET-CT diagnostic department, located mostly in the immediate vicinity of the production centre, each employee has been trained for the implementation of all procedures, so that, if necessary, they can perform any of the required production procedures. Here, however, the shift working system included only *Chemist* 3 and *Chemist* 4. Hence *Chemist* 4 as the only worker from the *chemists* group performs the dispensing of doses of ^18^F-FDG for the patients, and doing this only during the second shift. Figure 3 seems to confirm the assumption that specializing in carrying out specific activities optimizes radiation protection of personnel and helps reduce the doses of ionizing radiation.

It seems that the working system based on the combined radiopharmaceutical production procedures implemented in RPC II, compared to the RPC I should be a source of higher Hp(0.07) for the personnel performing only the procedure of ^18^F-FDG production or quality control of the radiopharmaceutical. The explanation of this fact seems simple: performing the same manipulations during a single procedure, every single day, results in higher efficiency and thus shortens the time performing the procedure. The only case when the recorded value of Hp(0.07) at all measurement points for the staff of the RPC I are higher in comparison with the RPC II concerns the cyclotron operating procedure. The tasks of chemists from the RPC I, in addition to the daily supervision of the proper functioning of the cyclotron and the replenishment of water enriched in ^18^O, includes the preparation of the tungsten containers containing the product ready to leave the centre. This is another example of an increased dose during the implementation of a greater number of procedures using the radiopharmaceutical (even if the radiopharmaceuticals are covered by tungsten).

Dispensing doses of ^18^F-FDG for patients according to the value specified by the medical doctor in a second production centre (RPC II) is also performed by *chemists* (two persons). Hand exposure of both employees looks different, as a consequence of differences relating to the way they work. *Chemist* 4 uses both hands when dispensing doses of ^18^F-FDG for the patient, as shown in particular in points 1–5 (Fig. 7). In the case of *Chemist* 3, if it is possible to perform the same procedure by using only one hand, the procedure is carried out just like that. This way of working reliably minimizes the exposure of the non-dominant hand. However, it affects the rate of actions and increases the exposure of the dominant hand. The data for *Chemist* 3 and 4 during the dispensing of doses of ^18^F-FDG for patients was also compared with the data from another diagnostic department, where this procedure is realized by a physicist. The results are shown in Fig. 8.

The procedure of filling up the syringe ^18^F-FDG with activity occurs automatically. Similarities also arise in which fingertips are the most exposed: the thumb and index finger of the right hand while performing this procedure. Despite these similarities, the difference between the Hp(0.07)/A for the thumb and index finger of the right hand for employees RPC II and MDC is, on average, three orders of magnitude. These differences can be explained by an individualization of manipulations and, above all, the time it takes to perform these.

Measurements performed in both centres also allow us to determine which of the activities/procedures carried out by the staff of both units contributes the most to the recorded dose. In the case of the RPC I, the greatest contribution to the dose recorded by TLD placed on the fingertips (80% for the left hand, and 89% for the right hand) is by the quality control of the radiopharmaceutical. The case of RPC II is similar. Here, the percentage of radiopharmaceutical quality control procedures for the left hand is over 96%; for the right hand, it is 83%.

## Conclusions

The measurements performed allow us to reach the following conclusions. The work system undoubtedly affects the level of recorded doses; specialization in performing specific activities/procedures shortens the time of performed procedures, which in turn results in lower doses. Automatic production of ^18^F-FDG promotes the optimization of radiation protection of personnel. Combining automatically performed production procedures with fragments of manual activities performed as part of the quality control of the radiopharmaceutical results in increased of hand exposure.

The highest ionizing radiation doses have been received by the staff during the ^18^F-FDG quality control. Hand exposure of nurses performing the administration of radiopharmaceuticals to the patient is different from the exposure of the hands of the chemists who dispensed the doses of ^18^F-FDG for patients. The source of lowest doses on the hands are the cyclotron operating procedure and the ^18^F-FDG production, provided that these procedures can’t be combined with other production procedures.

The maximum estimated annual Hp(0.07) for *chemists* from the quality control department employed in RPC I does not exceed 445 mSv. For the *physicists* from RPC I, it will not exceed 11 mSv. In the case of workers employed in RPC II, the maximum annual equivalent dose was estimated in the group of *chemists*—512 mSv. In the group of *nurses* it will reach up to 27% of the annual limit, while in the group of *physicists*, it will not exceed 0.6% of the annual dose limit, which is 500 mSv [12].
